# Supplementary material for: Point-to-point stabilized optical frequency transfer with active optics
Source: Nat Commun. 2021 Jan 22;12:515. doi: 10.1038/s41467-020-20591-5 (PMC7822849; doi:10.1038/s41467-020-20591-5)
Supplement: Supplementary file 1 — Supplementary Information [file 41467_2020_20591_MOESM1_ESM.pdf]

Supplementary Material: Point-to-Point Stabilized Optical Frequency Transfer with Active Optics

Benjamin P. Dix-Matthews[*,1,2], **Sascha W. Schediwy**[1,2], **David R. Gozzard**[1,2], **Etienne Savalle**[3], **François-Xavier Esnault**[4], **Thomas Lévèque**[4], **Charles Gravestock**[1], **Darlene D'Mello**[1], **Skevos Karpathakis**[1], **Michael Tobar**[2], **and Peter Wolf**[3]

[1]International Centre for Radio Astronomy Research, The University of Western Australia, Perth, Australia
[2]Australian Research Council Centre of Excellence for Engineered Quantum Systems, The University of Western Australia, Perth, Australia
[3]SYRTE, Observatoire de Paris, Université PSL, CNRS, Sorbonne Université, LNE, Paris, France
[4]Centre National d'Études Spatiales (CNES), Toulouse, France
[*]benjamin.dix-matthews@research.uwa.edu.au

Note 1: Phase noise measurement verification

Phase noise measurements in this work were performed by both a Microsemi 3120A Phase Noise Test Probe and an Ettus X300 Software Defined Radio. The Microsemi 3120A is a purpose-built phase/frequency noise measurement device, but is not able to handle large changes in signal amplitude. Performing a direct I/Q demodulation on the Ettus enabled us to continue making accurate and precise phase noise measurements in spite of large variations in signal amplitude and collect raw data time series at 200 kHz sampling for up to an hour. But the noise performance of the Ettus is less well known than the Microsemi, so to ensure compatibility between the measurements from the two devices, concurrent measurements of link phase noise by the two devices, shown in supplementary Fig. 1, were made and show that the two devices agree on the measured phase noise. Above a few tens of Hz the Ettus and Microsemi are in almost exact agreement. Below these frequencies some discrepancy between the Ettus and Microsemi results can be seen, possibly as a result of the Microsemi's behavior when trying to measure signals with large amplitude fluctuations. The discrepancy grows to a maximum difference of a factor of 2 at 1 Hz. Additionally, the noise floor of the Ettus was tested by measuring the phase noise of a Rigol DG-4102 synthesizer at 1 MHz with the same signal power (-15 dBm) as used in the field experiment. Both the Ettus and the synthesizer were referenced to the same 10 MHz. The phase noise measured by the Ettus is shown in supplementary Fig. 1. Note that the same linear phase drift of $0.141\,\mathrm{rad\,s^{-1}}$ was observed in the experimental phase data (see main text). This indicates that the noise floor of the Ettus was not dominating the measurement.

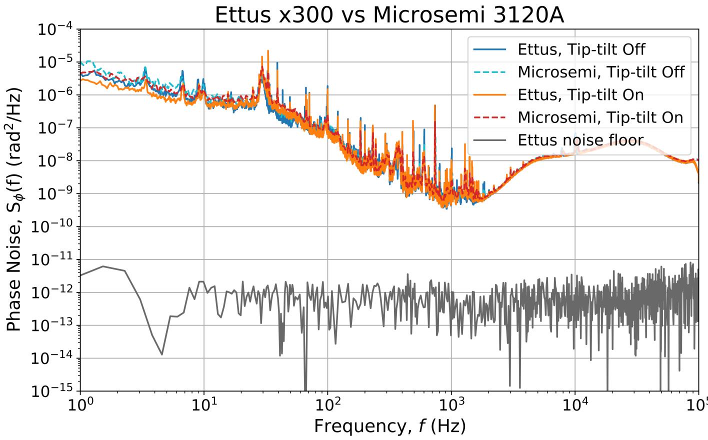

Figure 1. Concurrent measurements of 265 m free-space link phase noise using an Ettus X300 (solid blue, tip-tilt off; solid orange, tip-tilt on) and a Microsemi 3120A (dashed cyan, tip-tilt off; dashed red, tip-tilt on). The Ettus and Microsemi are in very close agreement across the Microsemi's frequency range. The noise floor of the Ettus (gray) was obtained by measuring the phase noise of a low noise synthesizer at 1 MHz.

Note 2: Noise contributions in parallel compensated links

Here, we estimate the noise floor of the phase noise measurements presented in the Results section. The aim is to estimate the phase stabilization noise floor caused by atmospheric noise and laser frequency noise when compensating the two links.

2.1 Differential noise PSD

For a noise process $x(t)$ that is stationary and characterized by a PSD $S_x(f)$, the PSD of the linear combination $z(t) \equiv \sum a_i x(t-T_i)$ (where a_i are arbitrary real constants) can be derived from first principles (see e.g. Duchayne[1] p.227) and is given by

$$S_z(f) = \left(\sum_i a_i^2 + \sum_i \sum_{j \neq i} a_i a_j \cos(2\pi f (T_i - T_j)) \right) S_x(f). \tag{1}$$

2.2 A single compensated link

Assume a compensated link (either of the two in Fig. 1 of main article) where a laser signal is frequency shifted by a transmission AOM before being injected into the link.

The frequency of the laser signal is $\nu_{\mathrm{L}}(t) = \nu_{\mathrm{L}} + \Delta\nu_{\mathrm{L}}(t)$ where $\Delta\nu_{\mathrm{L}}(t)$ is the laser frequency noise. The transmission AOM shifts the optical frequency by a nominal frequency (ν_{tr}) which is varied by $\Delta\nu_{\mathrm{tr}}(t)$ in order to suppress link noise. $\Delta\nu_{\mathrm{tr}}(t)$ is ideally equal but opposite to the frequency shift that the signal will experience during propagation through the link ($\delta\nu(t)$). At the remote site, the optical signal is passed through a static anti-reflection AOM (ν_{ar}) before being passed out of the remote site. We will define t as the instant the signal is passed out of the remote site. The laser signal coming out at the remote site has frequency $\nu_{\mathrm{out}}(t)$.

$$\nu_{\mathrm{out}}(t) = \nu_{\mathrm{L}} + \Delta\nu_{\mathrm{L}}(t-T) + \nu_{\mathrm{tr}} + \Delta\nu_{\mathrm{tr}}(t-T) + \nu_{\mathrm{ar}} + \delta\nu(t), \tag{2}$$

where T is the propagation time through the link.

The frequency shift $\delta\nu(t)$ is compensated by measuring, at the local end, the beat-note between $\nu_{\mathrm{L}}(t)$ and a return signal ($\nu_{\mathrm{ret}}(t)$) that went through the AOM twice (for simplicity we will ignore the remote site anti-reflection AOM, ν_{ar}, and the nominal transmission AOM frequency, ν_{tr}, applied by the transmission AOM):

$$\begin{aligned} \nu_{\mathrm{L}}(t) - \nu_{\mathrm{ret}}(t) &= \nu_{\mathrm{L}}(t) - [\nu_{\mathrm{L}}(t-2T) + \Delta\nu_{\mathrm{tr}}(t-2T) + \delta\nu(t-T) + \delta\nu(t) + \Delta\nu_{\mathrm{tr}}(t)] \\ &= \Delta\nu_{\mathrm{L}}(t) - [\Delta\nu_{\mathrm{L}}(t-2T) + \Delta\nu_{\mathrm{tr}}(t-2T) + \delta\nu(t-T) + \delta\nu(t) + \Delta\nu_{\mathrm{tr}}(t)] \end{aligned} \tag{3}$$

The phase-locked loop (PLL) [1] ensures that the beat-note signal (3) is zero, which means

$$\Delta\nu_{\mathrm{tr}}(t) + \Delta\nu_{\mathrm{tr}}(t-2T) = (\Delta\nu_{\mathrm{L}}(t) - \Delta\nu_{\mathrm{L}}(t-2T)) - (\delta\nu_{\mathrm{L}}(t-T) + \delta\nu_{\mathrm{L}}(t)). \tag{4}$$

We linearize the expression by a Taylor expansion of the left side leading to a differential equation:

$$\Delta\nu_{\mathrm{tr}}(t) - T\Delta\dot{\nu}_{\mathrm{tr}}(t) \simeq \frac{1}{2}(\Delta\nu_{\mathrm{L}}(t) - \Delta\nu_{\mathrm{L}}(t-2T)) - (\delta\nu(t-T) + \delta\nu(t)), \tag{5}$$

where we neglect higher order terms in the Taylor expansion. The general solution of the differential equation is

$$\Delta\nu_{\mathrm{tr}}(t) \simeq Ce^{t/T} + \frac{1}{2}(\Delta\nu_{\mathrm{L}}(t) - \Delta\nu_{\mathrm{L}}(t-2T)) - (\delta\nu(t-T) + \delta\nu(t)), \tag{6}$$

where C is an integration constant. As our loop is closed (the AOM frequency does not diverge) we have $C = 0$. Substituting that into (2) we finally get

$$\nu_{\mathrm{out}\,out}(t) = \nu_{\mathrm{L}} + \Delta\nu_{\mathrm{L}}(t-T) + \delta\nu(t) + \frac{1}{2}(\Delta\nu_{\mathrm{L}}(t-T) - \Delta\nu_{\mathrm{L}}(t-3T)) - (\delta\nu(t-2T) + \delta\nu(t-T)). \tag{7}$$

[1] We assume that the delay in the PLL is negligible with respect to T.

As one would expect, the expression reduces to $\nu_{\rm out}(t) = \nu_L + \Delta\nu_{\rm L}(t)$ when laser and link noise vary slowly with respect to T, i.e. the output signal is a copy of the input signal in spite of the presence of link noise.

Applying (1) to the laser noise contributions gives

$$S_{\rm out}(f) = S_{\Delta\nu}(f) + \frac{3}{2}\Big(1 - \cos(4\pi f T)\Big) S_{\Delta\nu}(f). \tag{8}$$

The second term quickly vanishes for $f \ll 1/T$ and we just have the input laser noise at the output. At approximately $f \geq 1/T$ the noise is increased by the compensation by a frequency dependent term that oscillates between 0 and $3S_{\Delta\nu}(f)$.

For the link noise, we obtain also from (1)

$$S_{\rm out}(f) = \Big(\frac{3}{2} - \frac{1}{2}\cos(2\pi f T) - \cos(4\pi f T)\Big) S_{\delta\nu}(f), \tag{9}$$

which, as expected, cancels completely when $f \ll 1/T$.

The total noise PSD of the output signal is the sum of (8) and (9).

2.3 Two parallel compensated links

We now consider two parallel compensated links A and B with delays T_A and T_B that are fed by the same input laser (see Fig. 1 of main article). We will be interested in the difference $D\nu_{\rm out}(t) \equiv \nu_{{\rm out}A}(t) - \nu_{{\rm out}B}(t)$, which is measured in the experiment. Each link is affected by the same laser noise $\Delta\nu_{\rm L}(t)$, but by different link noises $\delta\nu_A(t)$ and $\delta\nu_B(t)$.

For the laser noise applying (7) we directly have

$$\begin{aligned} D\nu_{\rm out}(t) &= \frac{3}{2}\Delta\nu_{\rm L}(t - T_A) - \frac{1}{2}\Delta\nu_{\rm L}(t - 3T_A) \\ &- \frac{3}{2}\Delta\nu_{\rm L}(t - T_B) + \frac{1}{2}\Delta\nu_{\rm L}(t - 3T_B). \end{aligned} \tag{10}$$

When the two links have the same delay ($T_A = T_B$) the laser noise cancels exactly. When they are different one can apply (1) to calculate the overall effect.

We assume that the link noise is uncorrelated between the two links, and thus the link PSDs given by (9) simply add.

The final result is then

$$\begin{aligned} S_{D\nu}(f) &= S_{\Delta\nu_{\rm L}}(f)\Bigg(5 - \frac{9}{2}\cos(2\pi f \Delta T) - \frac{1}{2}\cos(6\pi f \Delta T) \\ &- 6\Big(\cos(4\pi f T_A) + \cos(4\pi f T_B) + \cos(2\pi f (T_A + T_B))\Big)\sin^2(\pi f \Delta T)\Bigg) \\ &+ S_{\delta\nu A}(f)\Big(\frac{3}{2} - \frac{1}{2}\cos(2\pi f T_A) - \cos(4\pi f T_A)\Big) \\ &+ S_{\delta\nu B}(f)\Big(\frac{3}{2} - \frac{1}{2}\cos(2\pi f T_B) - \cos(4\pi f T_B)\Big). \end{aligned} \tag{11}$$

where $\Delta T \equiv T_A - T_B$. As expected from (10), the laser noise contribution vanishes for $T_A = T_B$. In the limit when $f \ll 1/T$ the link noise contributions vanish, and (11) can be approximated as

$$S_{D\nu}(f) \simeq S_{\Delta\nu_{\rm L}}(f)\, 36\pi^4 (T_A^2 - T_B^2)^2 f^4. \tag{12}$$

2.4 Noise floor estimation

To estimate the noise floor in our compensated link we need to evaluate (11) using estimates of the free space link noise $S_{\delta\nu A}(f)$, the fiber link noise $S_{\delta\nu B}(f)$, and the laser noise $S_{\Delta\nu_{\rm L}}(f)$. We assume that the fiber link noise is negligible with respect to the atmospheric noise and use the "unstabilized, tip-tilt off" measurement (see Fig. 2 of main article) as our estimate of the latter. For the laser noise we use the "typical" noise curve given by the manufacturer[2]. The two delays were estimated from delay measurements in the fiber and from local maps as $cT_A \approx 302$ m (265 m free space + 25 m fibers with refractive index $n = 1.45$ between the telescopes and the beam splitters), and $cT_B = n \times 715$ m. The result is shown in supplementary Fig. 2.

We note that above about 200 Hz our measured results are well explained by the combined effects of atmospheric turbulence and laser noise, at least in terms of the orders of magnitude. The residual noise of the compensated links is dominated by atmospheric noise between 200 Hz and 2 kHz, and laser noise takes over at higher frequencies. The slight discrepancy in the atmospheric effect is probably due to the fact that the uncompensated measurement (solid red in supplementary Fig. 2)

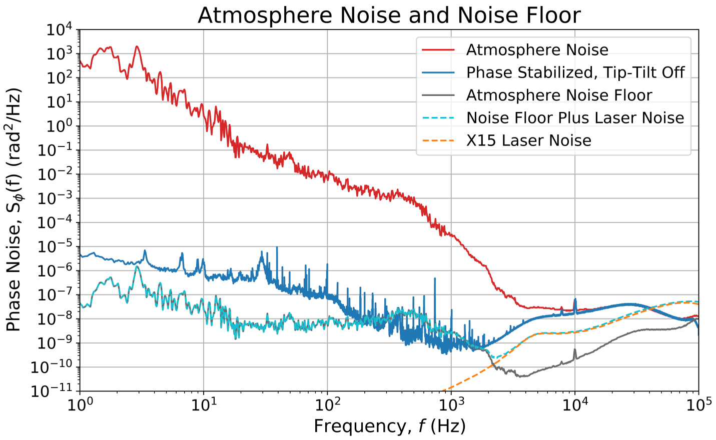

Figure 2. Estimate of the noise floor for the parallel compensated links. Red, free-running noise from Fig. 2 of main article dominated by atmospheric phase noise; blue, phase stabilized, tip-tilt off result from Fig. 2 of main article; gray, estimate of noise floor due to atmosphere; dashed cyan, atmospheric noise floor plus estimate of laser noise floor over the parallel links; dotted orange, estimate of laser noise floor.

was taken at a different time than the compensated one. The discrepancy at higher frequency is most likely due to the fact that the NKT X15 laser we used had more phase noise than the “typical" performance of that type of laser specified by the manufacturer in[2]. Nonetheless the similar shape and the fact that all measured curves on Fig. 2 of main article converge above 10 kHz comforts us in the hypotheses that laser noise is the common origin. The exception is the “system noise floor" shown in gray on Fig. 2 of main article for which two parallel fibers of equal length were used i.e. $T_A = T_B$ in (11) and laser noise cancels, as can be observed.

References

1. Loïc Duchayne, PhD thesis, Paris Observatory, 2008. https://tel.archives-ouvertes.fr/tel-00349882
2. Koheras Basik X15 specifications. https://www.nktphotonics.com/lasers-fibers/product/koheras-basik-low-noise-single-frequency-oem-laser-modules/
